# Physiological and Anthropometric Factors Associated With Spine Loading Estimates From Imaging‐Based Subject‐Specific Musculoskeletal Models

**DOI:** 10.1002/jsp2.70059

**Published:** 2025-04-11

**Authors:** Brett T. Allaire, Fjola Johannesdottir, Mary L. Bouxsein, Dennis E. Anderson

**Affiliations:** ^1^ Center for Advanced Orthopaedic Studies, Beth Israel Deaconess Medical Center Boston Massachusetts USA; ^2^ Department of Orthopaedic Surgery Harvard Medical School Boston Massachusetts USA

**Keywords:** computed tomography, musculoskeletal models, predictive analysis, principal component analysis, spine loading

## Abstract

**Background:**

Subject‐specific musculoskeletal models may be used to estimate spine loads that cannot be measured in vivo. Model generation methods may use detailed measurements extracted from medical imaging, but it may be possible to create accurate models without these measurements. We aimed to determine which physiological and anthropometric factors are associated with spine loading and should be accounted for in model creation.

**Methods:**

We created models of 440 subjects from the Framingham Heart Study Multi‐detector CT Study, extracting muscle morphology and spine profile information from CT scans of the trunk. Five lifting activities were simulated, and compressive and shear loading estimates were produced. We performed principal component analysis on the loading data from three locations in the spine, as well as univariate correlations between predictor variables and each principal component (PC). We identified multivariate predictive regression models for each PC and individual loading estimate.

**Results:**

A single PC explained 90% of the variability in compressive loading, while four PCs were identified that explained 10%–37% individually, 86% in total, of the variability in shear loading. Univariate analysis showed that body weight, BMI, lean mass, and waist circumference were most associated with the compression PC and first shear PC. Multivariate regression modeling showed predictor variables predicted 94% of the variability in the compression PC, but only 54% in the first shear PC, with body weight having the highest contribution. Additional shear PCs were less predictable. Level‐ and activity‐specific compressive loading was predicted using a limited set of physiological and anthropometric factors.

**Conclusions:**

This work identifies easily measured characteristics, particularly weight and height, along with sex, associated with subject‐specific loading estimates. It suggests that compressive loading, or models to evaluate compressive loading, may be based on a limited set of anthropometric attributes. Shear loading appears more complex and may require additional information not captured in the set of factors we examined.

## Graphical abstract

Utilizing CT scans from a population‐based cohort, we identified several physiological and anthropometric characteristics that were associated with loading estimates from imaging‐based musculoskeletal models. Shear loading is more complex than compressive loading and is not easily explained by basic clinical factors.

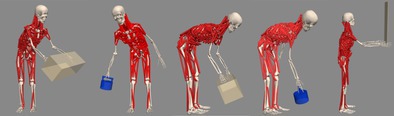

## 1 Introduction

Mechanical demands on the musculoskeletal structure of the spine are intimately interconnected with spinal conditions such as spinal deformity [1], vertebral fractures [2], spinal stenosis [3], and back pain [4, 5]. Thus, the assessment of in vivo mechanical demands is of interest for a better understanding of the risks of, and clinical interventions for, a variety of spine conditions. However, methods for directly measuring in vivo demands, such as instrumented implants and intervertebral disc pressure, are invasive and impractical for general use [6, 7]. Musculoskeletal modeling provides a noninvasive method to estimate spine loads [8, 9], and various computational musculoskeletal modeling approaches have been created and validated for this purpose [10, 11, 12, 13].

Musculoskeletal models can be personalized to widely varying degrees, from simple scaling to match subject height and weight to incorporating multiple measurements taken from medical imaging. A few studies have quantified the understanding from basic biomechanical principles that body weight and posture are related to spinal loads, with weight having greater importance than age, height, and sex [14, 15, 16, 17]. Further incorporating CT‐based measurements of spine curvature and muscle geometry can alter estimated spinal loads by up to 50% [18]. While it is clear that model personalization matters, it remains largely unknown whether accurate models can be generated in the absence of such imaging measurements. We propose that accurate subject‐specific models can be generated to evaluate spine loading with appropriate, easily obtained patient characteristics. Here, we aim to address the question of which physiological and anthropometric characteristics are associated with spine loading estimates from image‐based subject‐specific modeling and thus should be accounted for during model creation. First, we will determine which physiological and anthropometric characteristics are associated with spine loading. Then, we will test the ability of the identified factors to predict spine loading by employing regression modeling.

## 2 Methods

### 2.1 Participants

We evaluated previously collected data of 55 men and 55 women from each of four age decades (40–49, 50–59, 60–69, and 70–79) from the Framingham Heart Study Multi‐detector CT Study, totaling 440 subjects [19]. Subjects were eligible if they had clinical exam data and a dual‐energy x‐ray absorptiometry (DXA) scan within 1 year of their CT scan. In the end, one subject was excluded because of missing select clinical variables, which resulted in a final cohort of 219 men and 220 women. This study was approved by the institutional review boards of Boston University Medical Center (which reviews the Framingham Heart Study) and Beth Israel Deaconess Medical Center.

### 2.2 Spine Loading Estimates

We measured trunk muscle size and position in previously collected CT scans of the abdomen and thorax (Analyze, Biomedical Imaging Resource) and adjusted modeled muscle geometric and strength properties based on these data. We also incorporated sagittal spine alignment measured from lateral CT scout images (SpineAnalyzer 4.0, Optasia Medical) [20, 21]. Using these data along with subject sex, height, and weight, we created subject‐specific musculoskeletal models in OpenSim (Version 4.3) software using methods described previously [18, 22, 23]. The resulting full‐body models included upper and lower extremities, a fully articulated thoracolumbar spine with 51 intervertebral rotational degrees of freedom, and 620 musculotendon actuators.

**FIGURE 1:**
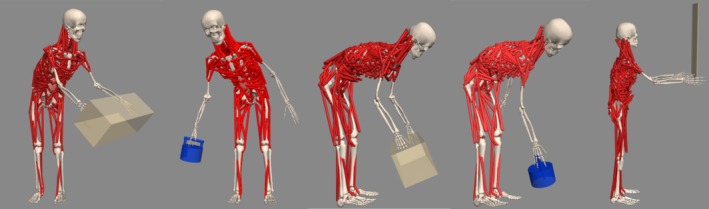
Tasks simulated in OpenSim. From left to right: Axial lift, lateral lift, flexion lift with two hands, flexion lift with one hand, and opening a window. Weight lifted is set at 10% of body weight.

With each subject‐specific model, we evaluated spine loading for five different quasi‐static activities involving multi‐axial spine movement for which we had previously collected motion analysis data. These activities are: axial twist holding a box in two hands (Axl); lateral bend lifting a box with the right hand (Lat); flexing picking up a box with two hands (Sag); flexing picking up a weight in the right hand (Sag1h); and simulating opening a window (Win) which involved raising a 5 N weight at arm's length from the body (Figure 1). Box weight for the lifting activities was set at 10% of the subject's weight. We used separately measured kinematics from 10 similarly aged subjects who completed these tasks in a motion analysis laboratory and defined the static pose for analysis as the moment of peak loading during the movement [22]. All 10 kinematic inputs for each activity were used to perform static optimization analyses for each subject. The static optimization algorithm minimized the sum of musculotendon actuator activations cubed. We have previously shown the validity of using separately measured kinematics in this manner for estimating spine loading [24]. The primary outcomes were compressive and resultant shear loads at each vertebral level, averaged across the 10 kinematic inputs. Loads at L3, T12, and T8 were included for further analyses.

### 2.3 Physiological and Anthropometric Factors

Framingham Heart Study participants underwent regularly scheduled clinical exams. Physiological and anthropometric factors were extracted from clinical exam data at the exam nearest to the CT scan or from the CT scan itself. The characteristics that were considered as predictors for spine loading included age, sex, height (Ht), weight (Wt), waist circumference (Waist), body mass index (BMI), thoracic kyphosis, lumbar lordosis, whole body fat mass (TotFat) and whole‐body lean mass (TotLean). Waist was measured from a cross‐sectional CT image at the level of the umbilicus, and body composition was assessed by DXA. Thoracic kyphosis (T4–T12) and lumbar lordosis (L1–L5) were calculated as Cobb angles from six‐point morphometry analysis of lateral CT scout images [25].

### 2.4 Statistical Analysis

Descriptive data are presented as mean ± standard deviation (SD). Pearson correlation was used to assess the association between loadings across activities and vertebral levels. Due to the multi‐collinearity between loading estimates, we performed principal component analysis (PCA) to transform the data set into a set of uncorrelated principal components (PCs). PCA was performed separately for compressive and shear loading estimates. Only PCs with eigenvalues > 1 were included in further analysis based on the Kaiser Rule [26]. We calculated the PCs loading matrix to interpret what loading estimates were most correlated with each PC, calculated as $\text{eigenvectors}\times \sqrt{\text{eigenvalues}}$. Univariate correlation between each predictor variable and PC was calculated, and multivariate predictive regression models were identified for each PC based on the Bayes information criterion (BIC). Nonlinearity between predictor variables and each PC was examined visually with scatter plots with loess fit superimposed. No nonlinearity was identified (results not shown). The relative importance of each factor in the predictive regression models was estimated by calculating the relative contribution of each physiological or anthropometric factor to the *R* ^2^ using a method that accounts for the dependence of orderings of predictors in the model, using the R package *relaimpo* [27]. Further, we verified that each individual spinal compressive loading estimate was associated with the factors that were identified through PCA. We fitted linear mixed models (LMMs) for each activity with vertebral level as repeated measurements. The data were split into a training set (75%, *n* = 331) and a test set (25%, *n* = 108). The LMMs were built on the training set, and then the performance of the models were estimated using the test set. The performance metrics that were calculated included the root mean square error (RMSE) in absolute unit (N) and percentage (%) of mean load as well as *R* ^2^, using the fixed effects from the LMMs. Such analysis was not performed for shear loading as the physiological and anthropometric factors we examined explained little of the variability (~27% of total variance for all PCs combined). Further, we plotted load vs. predicted load and assessed the error with Bland and Altman plots. Statistical analyses were performed using R 4.2.2 (R Foundation for Statistical Computing), and *p* values < 0.05 were considered significant.

## 3 Results

### 3.1 Cohort Characteristics

The average age of the cohort was 59.4 years (SD 11.6 years), with an average BMI of 28.1 (4.6) (Table 1). The cohort by race/ethnicity included 9% Asian, 8% Black, 14% Hispanic, 68% White, and 1% Other/Mixed participants.

**TABLE 1:** Population characteristics.

	Total, *n* = 439	Men, *n* = 219	Women, *n* = 220
Age (years)	59.4 (11.6)	59.2 (11.6)	59.5 (11.53)
Race (*n*, %)			
Asian	40 (9%)	19 (8.7%)	21 (9.5%)
Black	36 (8%)	13 (5.9%)	23 (10.5%)
Hispanic	61 (14%)	25 (11.4%)	36 (16.4%)
White	298 (68%)	161 (73.5%)	137 (62.3%)
Mixed/other	4 (1%)	1 (0.5%)	3 (1.4%)
Height (cm)	167.4 (9.8)	174.2 (7.3)	160.5 (6.7)
Weight (kg)	79.2 (16.6)	87.4 (14)	71 (14.9)
BMI (kg/m^2^)	28.1 (4.6)	28.7 (4)	27.5 (5.1)
Total body fat (g)	27 140 (9112)	25 573 (8101)	28 700 (9790)
Total lean mass (g)	47 778 (11319)	56 770 (7319)	38 827 (6449)
Waist circumference (cm)	98.58 (12.48)	101.84 (10.64)	95.33 (13.32)
Kyphosis (degrees)	26.74 (8.63)	25.58 (8.68)	27.89 (8.46)
Lordosis (degrees)	−15.98 (9.19)	−14.59 (8.73)	−17.37 (9.43)

*Note:* mean (SD).

### 3.2 Association Between Subject Characteristics and Spine Loading

**FIGURE 2:**
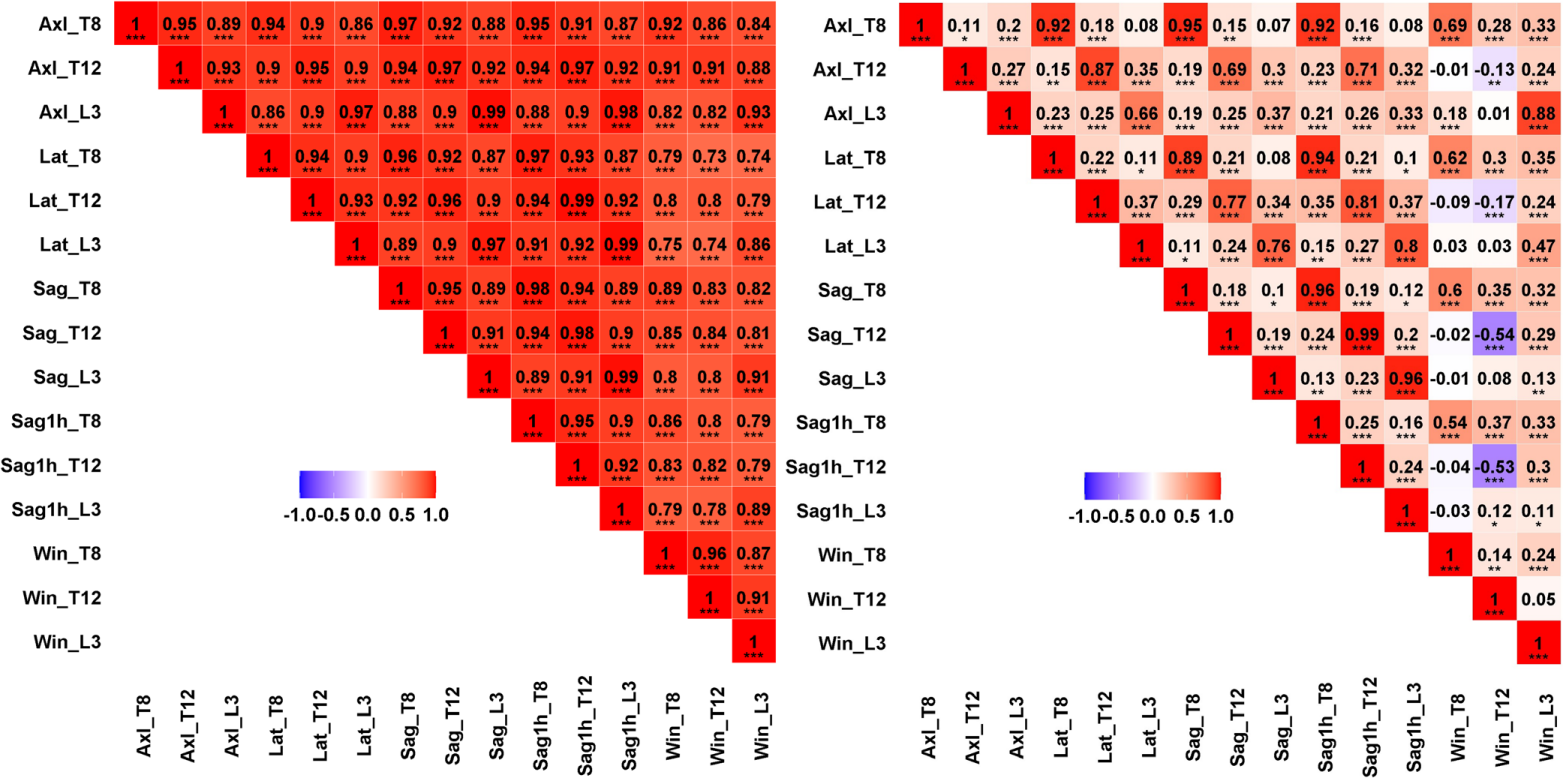
Correlation between compressive loadings across levels and activities (left), and correlation between shear loadings across levels and activities (right). ***p < 0.001, **p < 0.01, *p < 0.05.

For all activities besides opening a window (Win), compressive loading was highest at L3 (*p* < 0.001) (Table 2). Spine loading estimates were correlated across levels and activities (Figure 2), particularly for compressive loading, where the correlation ranged from 0.74 to 0.99 (*p* < 0.001, Figure 2). For shear loading, the estimates ranged from being not correlated to being correlated as high as 0.99 (Figure 2). Overall, the correlation was higher across activities at the same level than between different levels within an activity (Figure 2).

**TABLE 2:** Summary of loading data.

	Total, *n* = 439		Men, *n* = 219		Women, *n* = 220	
	Compressive load (N)	Shear load (N)	Compressive load (N)	Shear load (N)	Compressive load (N)	Shear load (N)
Axl_T8	1987 (461)	358 (111)	2274 (461)	383 (111)	1702 (366)	333 (100)
Axl_T12	3111 (628)	253 (102)	3507 (628)	258 (102)	2717 (502)	249 (100)
Axl_L3	3271 (728)	248 (150)	3700 (728)	250 (150)	2845 (596)	246 (132)
Lat_T8	988 (242)	221 (58)	1151 (242)	236 (58)	825 (170)	206 (50)
Lat_T12	1489 (295)	211 (66)	1681 (295)	225 (66)	1298 (228)	198 (62)
Lat_L3	1905 (446)	122 (71)	2194 (446)	139 (71)	1618 (328)	105 (68)
Sag_T8	1299 (334)	344 (90)	1533 (334)	373 (90)	1066 (232)	315 (77)
Sag_T12	2238 (490)	315 (124)	2571 (490)	317 (124)	1907 (361)	313 (114)
Sag_L3	2958 (670)	129 (112)	3385 (670)	151 (112)	2534 (518)	107 (93)
Sag1h_T8	967 (239)	273 (66)	1135 (239)	296 (66)	800 (164)	249 (58)
Sag1h_T12	1631 (346)	226 (88)	1859 (346)	231 (88)	1404 (265)	221 (82)
Sag1h_L3	2190 (495)	108 (80)	2501 (495)	127 (80)	1881 (386)	89 (70)
Win_T8	771 (144)	64 (35)	853 (144)	62 (35)	688 (118)	66 (33)
Win_T12	1046 (180)	87 (45)	1156 (180)	100 (45)	937 (145)	74 (42)
Win_L3	894 (163)	142 (54)	986 (163)	149 (54)	802 (137)	136 (52)

*Note:* Mean (SD).

Abbreviations: Axl, axial twist lift; Lat, lateral bend lift; Sag, sagittal bend lift; Sag1h, sagittal one‐handed (right) lift; Win, opening a window.

**FIGURE 3:**
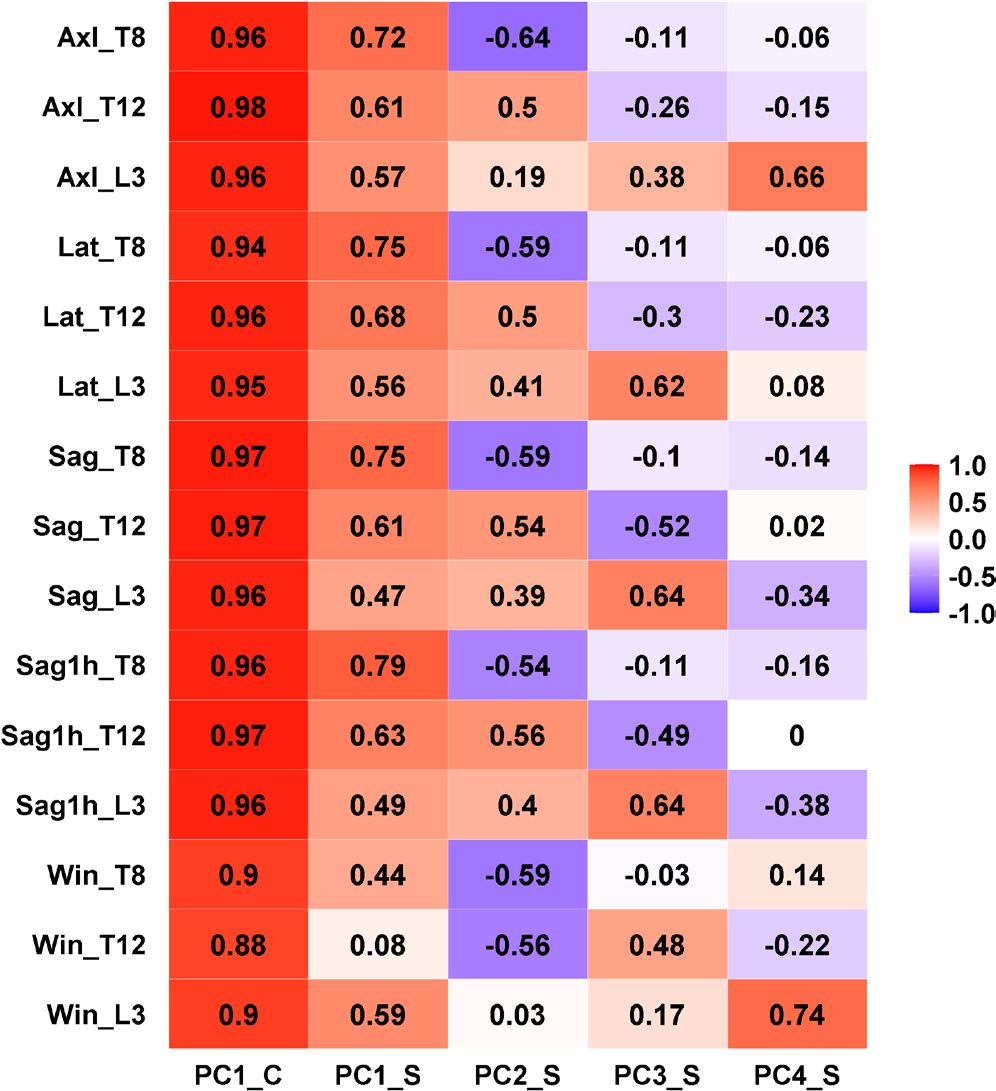
Principal components (PC) loading matrix. C, compression; S, shear.

For compressive loading, one PC (PC1_C) explained 90% of the variability in the data. All compressive loading estimates were highly correlated with PC1_C (*r* = 0.88–0.96, Figure 3). Four PCs were identified for shear loading (PC1_S, PC2_S, PC3_S, and PC4_S) that explained 10%–37% individually and 86% in total of the variability in the data (Table 3). PC1_S was most associated with shear loading at T8, PC2_S was positively associated with shear loading at T12 and negatively associated with T8, PC3_S was associated with loading at L3 for lateral and flexion lifts, and finally, PC4_S was most correlated with loading at L3 during axial lift and opening a window (Figure 3).

**TABLE 3:** Best predictive model for each principal component (PC).

	PC1_C	PC1_S	PC2_S	PC3_S	PC4_S
%Variance explained	90%	37%	24%	16%	10%
Age		X	X		
Sex	X	X		X	
Height	X		X	X	
Weight	X	X			X
Lordosis	X	X			X
Kyphosis	X		X	X	X
BMI					
Total lean mass					
Total body fat					
Waist circumference					
Adjusted *R* ^2^	0.94	0.54	0.17	0.10	0.13

*Note:* X indicates that a variable is included in the model.

Abbreviations: C, compression; S, shear.

**FIGURE 4:**
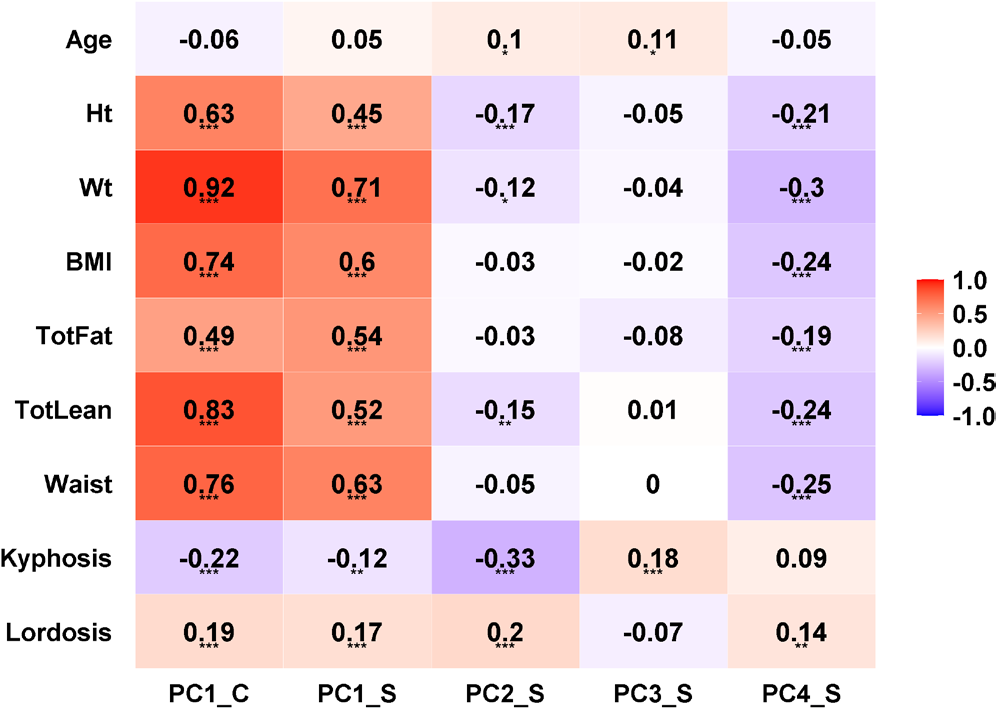
Correlation between principal components (PC) and patient‐specific clinical measurements. ***p < 0.001, **p < 0.01, *p < 0.05.

In univariate correlation analyses, we found that body weight, BMI, lean mass, and waist circumference were associated with both PC1_C (*r* = 0.74–0.92, *p* < 0.001, Figure 4) and with PC1_S (*r* = 0.52–0.71, *p* < 0.001). PC2_S showed the highest correlation with spinal curvature (both kyphosis and lordosis). In a multivariate regression model, physiological and anthropometric variables (sex, height, weight, lordosis, and kyphosis) predicted 94% of the variability in PC1_C (Table 3). Physiological and anthropometric variables predicted 54% of the variability in PC1_S, but only explained 10%–17% of the variability in PC2_S–PC4_S (Table 3). Body weight contributed the most to explaining the variability in PC1 for both compressive and shear loading, explaining 61% and 87% of the variance in the predictive model for PC1_C and PC1_S, respectively.

### 3.3 Prediction of Spinal Compressive Loading

**FIGURE 5:**
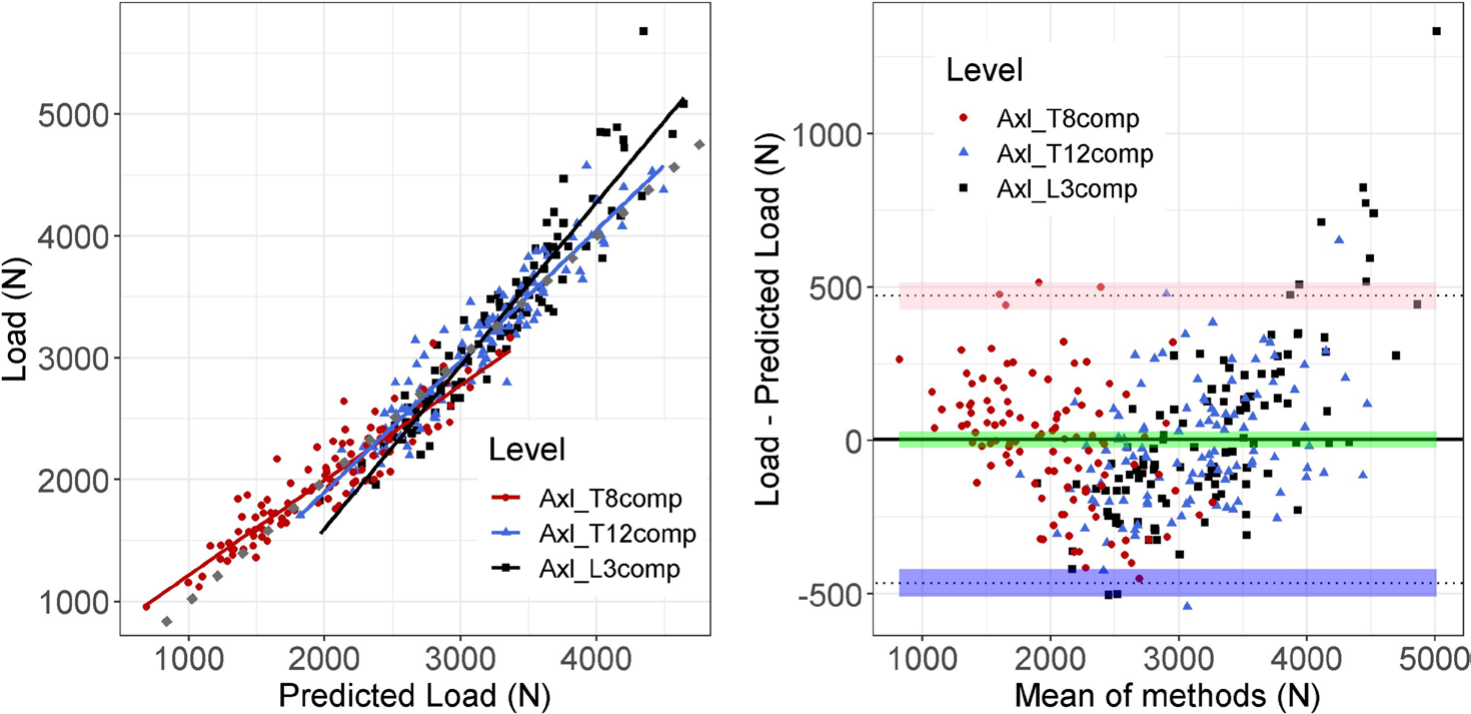
Predicted compressive loading vs. compressive loading during axial lifting (left), gray dot line is superimposed line of unity. Bland and Altman plot for prediction of compressive loading during axial lifting (right).

Subject characteristics and spine loads were similar between the training and test sets of subjects (*p* > 0.67). Compressive loading for all five activities and across vertebral levels was predicted well from subject sex, height, weight, lumbar lordosis, and thoracic kyphosis (Table 4). The percent RMSE ranged from 6.4% to 12.2% for all activities and across levels (Table 4), while *r* ^2^ ranged from 0.71 to 0.91 for the same activities. The agreement between loading estimates and predicted loading was good and without a bias overall (Figure 5 for axial lifting; see Supporting Information S1 for other activities). However, there appeared to be a tendency for under‐prediction of larger loads (e.g., > 4000 N) for level L3 (Figure 5).

**TABLE 4:** Performance of predictive models to predict compressive loading.

Activity		RMSE (N)	RMSE (%)	*R* ^2^
Axial lift	T8	207	10.4	0.86
	T12	200	6.4	0.90
	L3	297	9.0	0.91
Lateral lift	T8	114	11.6	0.83
	T12	119	8.0	0.84
	L3	234	12.2	0.89
Flexion lift with two hands	T8	156	12.0	0.84
	T12	198	8.9	0.85
	L3	357	12.0	0.89
Flexion lift with one hand	T8	101	10.5	0.87
	T12	136	8.4	0.87
	L3	273	12.4	0.91
Opening a window	T8	63	8.2	0.79
	T12	93	8.9	0.71
	L3	71	7.9	0.83

*Note:* RMSE is root mean square error.

## 4 Discussion

We examined a range of easily obtained physiological and anthropometric measures for their relationship with estimated spine loading in a population‐based cohort. We found that such measures are predictive of compressive loading in multiple loading scenarios, particularly subject weight, height, and spinal curvature, with weight having the largest contribution. This is not surprising under basic biomechanical principles and agrees with previous literature showing weight is highly correlated with compressive loading and that weight is a more important factor than age, height, or sex [14, 15, 16, 17].

Ghezelbash et al. showed that key predictors of lower spine loading were trunk kinematics, weight lifted, hand position (e.g., shoulder‐to‐load distance), and body weight [17]. Here, we showed similar predictive values (*R* ^2^ and RMSEs) of the physiological and anthropometric factors we identified when predicting compressive loading in several realistic postures. Our focus was on intrinsic subject factors, and we similarly found that body weight was important. We also found contributions of lumbar lordosis and thoracic kyphosis, which were assessed here by radiographic means. Several studies have proposed means to estimate radiographic Cobb angles from nonradiographic measures, which could provide for the use of such prediction equations without imaging [28, 29]. Though we were not attempting to examine the effects of weight lifted or variations in kinematics here, we acknowledge that such activity‐specific factors are critical determinants of spine loading. Because of the specific nature of the activities tested, we do not propose to be able to predict or give estimates of compressive loading for activities other than those tested here.

Shear loading was more variable between vertebral levels and across activities. This suggests that different factors outside the specific set of characteristics we examined may influence shear loading. A previous study showed good predictability of shear loading, but only lower lumbar regions were examined (L4–L5 and L5–S1) [17]. Shear loading here was found to be less correlated between spinal levels than compressive loading, and shear loading at various locations may be sensitive to different factors and loading scenarios. Thus, situations where shear load prediction is important may call for the use of musculoskeletal modeling to capture these subtle interactions. Vertebral failure loads in shear can be much lower than in compression [30], and the risk of shear failure is an important aspect of injury risk assessments. Musculoskeletal modeling is advantageous because it yields shear loading in addition to compression and allows assessment of a wide range of activities. Future work may include testing more strenuous activities or targeting scenarios that produce higher shear loading.

This study has notable strengths but also key limitations that should be acknowledged. The musculoskeletal modeling approach we used has well‐known limitations, including the use of static optimization, which tends to underestimate muscle antagonistic activity [31, 32]. Additionally, it does not incorporate passive forces from intervertebral ligaments and discs, and the personalization of the spine and muscle was based on geometric measurements in CT images only, as accurate characterization of personalized mechanical properties from imaging remains difficult. Furthermore, curvature and muscle measurements were made from CT scans acquired in the supine position. Though it is important to note all CT scans were acquired using the same protocol, this likely affects measurements uniformly across all subjects. Nonetheless, the modeling approach has been validated for the estimation of spine loading and dynamic muscle activity [11, 33], and the data set included CT‐based subject‐specific models of 439 individuals from a population‐based cohort, a significant strength. While we were not able to measure kinematics in these 439 subjects of the Framingham cohort, we applied multiple measured kinematics from individuals of similar age performing the tasks, a method we have shown to produce reasonable estimates of spine loading [24] and a strength compared to prior studies using a single estimated pose for the assessment of spine loading in large cohorts [23, 34]. It should be noted that static peak loading outcomes, as found here, are approximately 20% less than the comparable dynamic loads [22], but this should not affect our conclusions as to the overall effects of intrinsic factors on spine loading outcomes. Finally, our set of activities, while limited, provides a sampling of possible normal daily lifting tasks, including nonsagittal motions, an important strength when considering the wide range of possible loading conditions for the spine. Overall, this study represents one of the most comprehensive evaluations of factors affecting spine loading performed to date, in a large sample from a population‐based cohort using a well‐validated musculoskeletal modeling approach. Thus, it provides unique insight into how intrinsic subject characteristics affect spine loading in adults.

In conclusion, we identified several easily measured factors that explain a large portion of the variability in vertebral compressive loading in a large population‐based cohort. Our findings suggest it may be possible to estimate vertebral compressive loading using a limited set of physiological and anthropometric factors. We found that shear loading is more complex and was less readily predicted by the limited set of factors we examined than compressive loading. This result, while not surprising, suggests that additional research is needed to identify other factors that influence shear loading along the spine. Overall, these results also suggest that musculoskeletal models of the spine accounting for a few key personal characteristics may prove sufficient for the prediction of compressive loading in most cases, while accurate assessment of shear loading may yet require higher fidelity modeling based on imaging or other future developments.

## Conflicts of Interest

The authors declare no conflicts of interest.

## Supporting information


**Data S1.** Supporting Information.
