# Supplementary material for: Physiological and Anthropometric Factors Associated With Spine Loading Estimates From Imaging‐Based Subject‐Specific Musculoskeletal Models
Source: JOR Spine. 2025 Apr 11;8(2):e70059. doi: 10.1002/jsp2.70059 (PMC11987705; doi:10.1002/jsp2.70059)
Supplement: Supplementary file 1 — Data S1. Supporting Information. [file JSP2-8-e70059-s001.docx]

**Supplementary Materials**


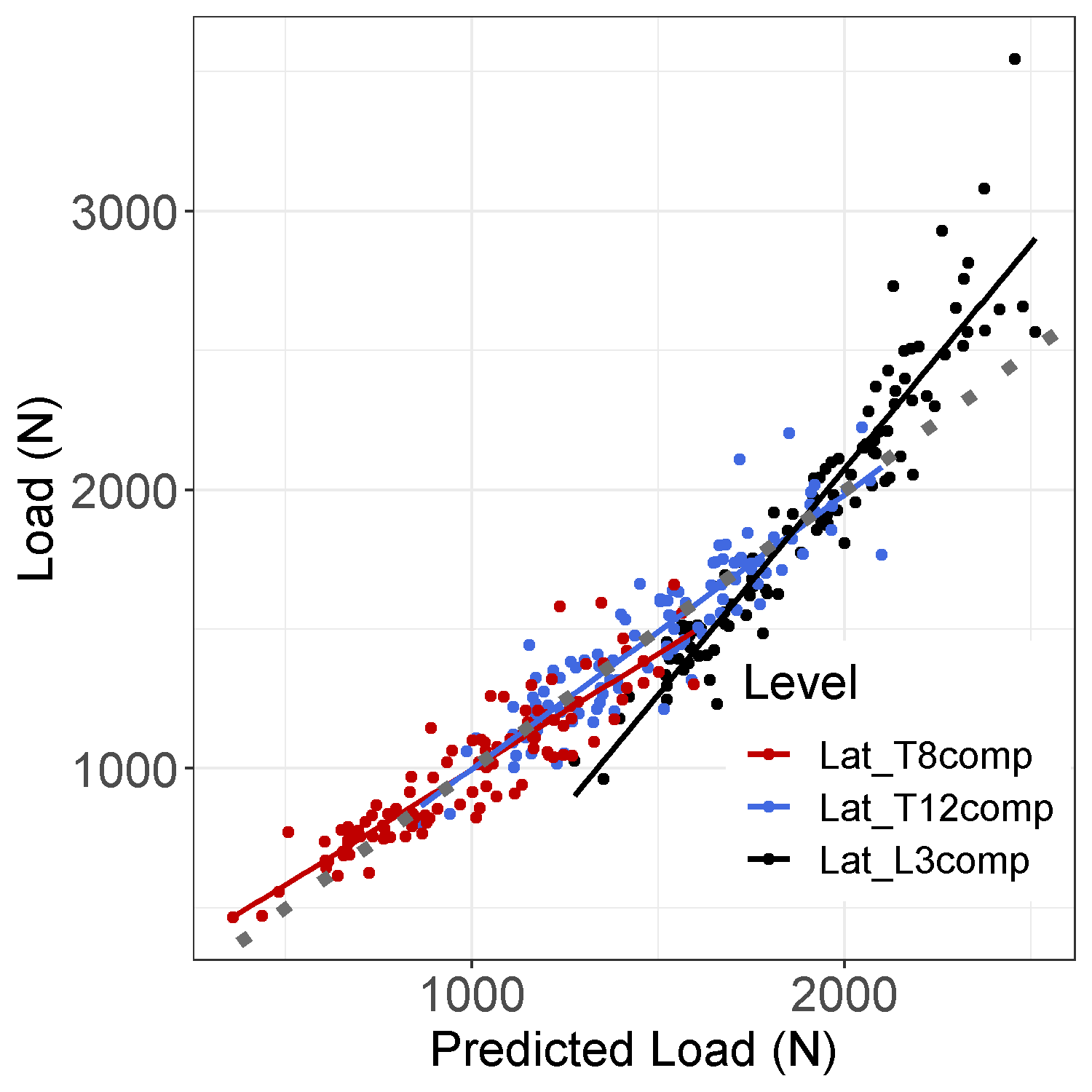

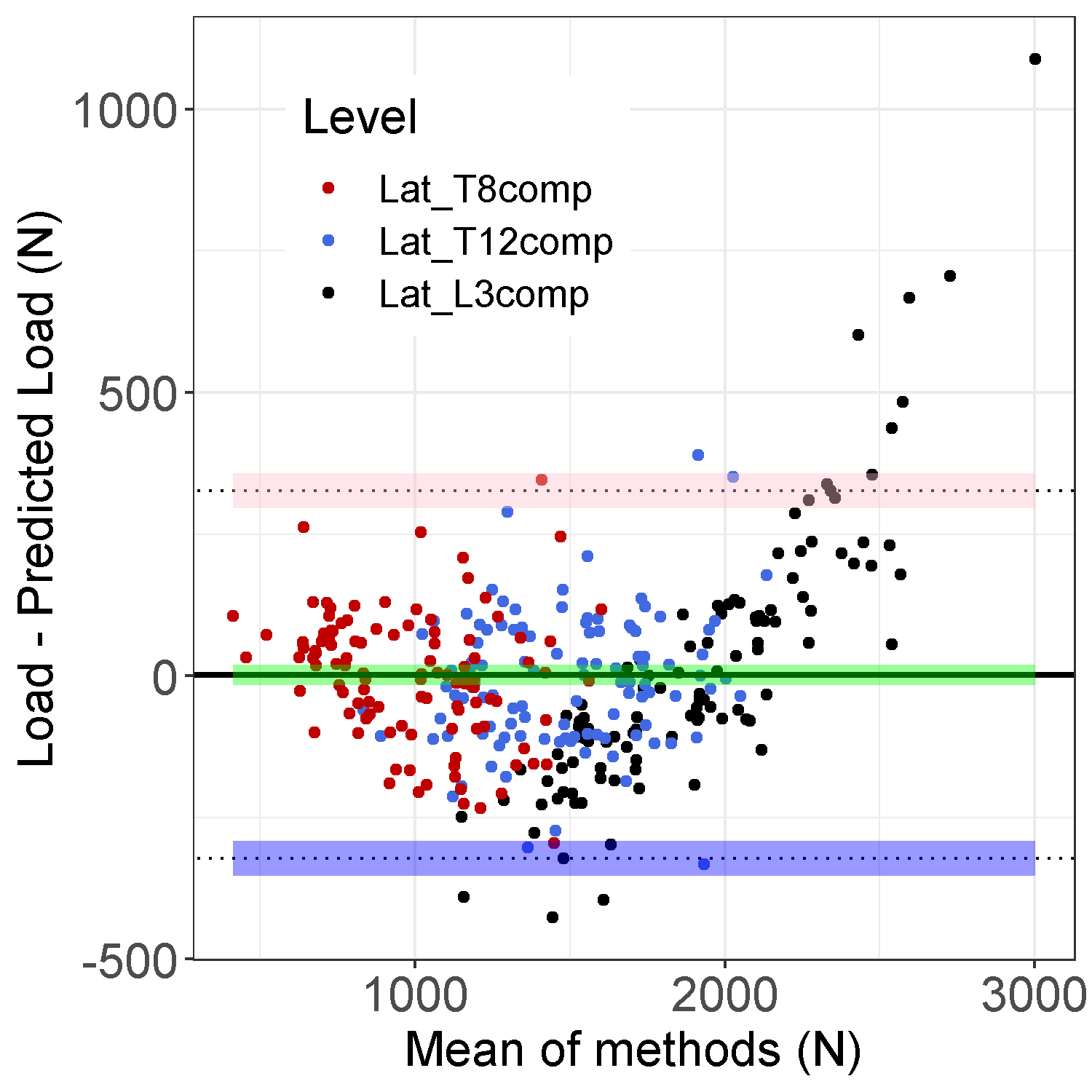


**Fig. S1**: A) Predicted compressive loading vs compressive loading during lateral bend lifting a box with the right hand, grey dot line is superimposed line of unity. B) Bland and Altman plot for prediction of compressive loading during lateral bend lifting a box with the right hand.


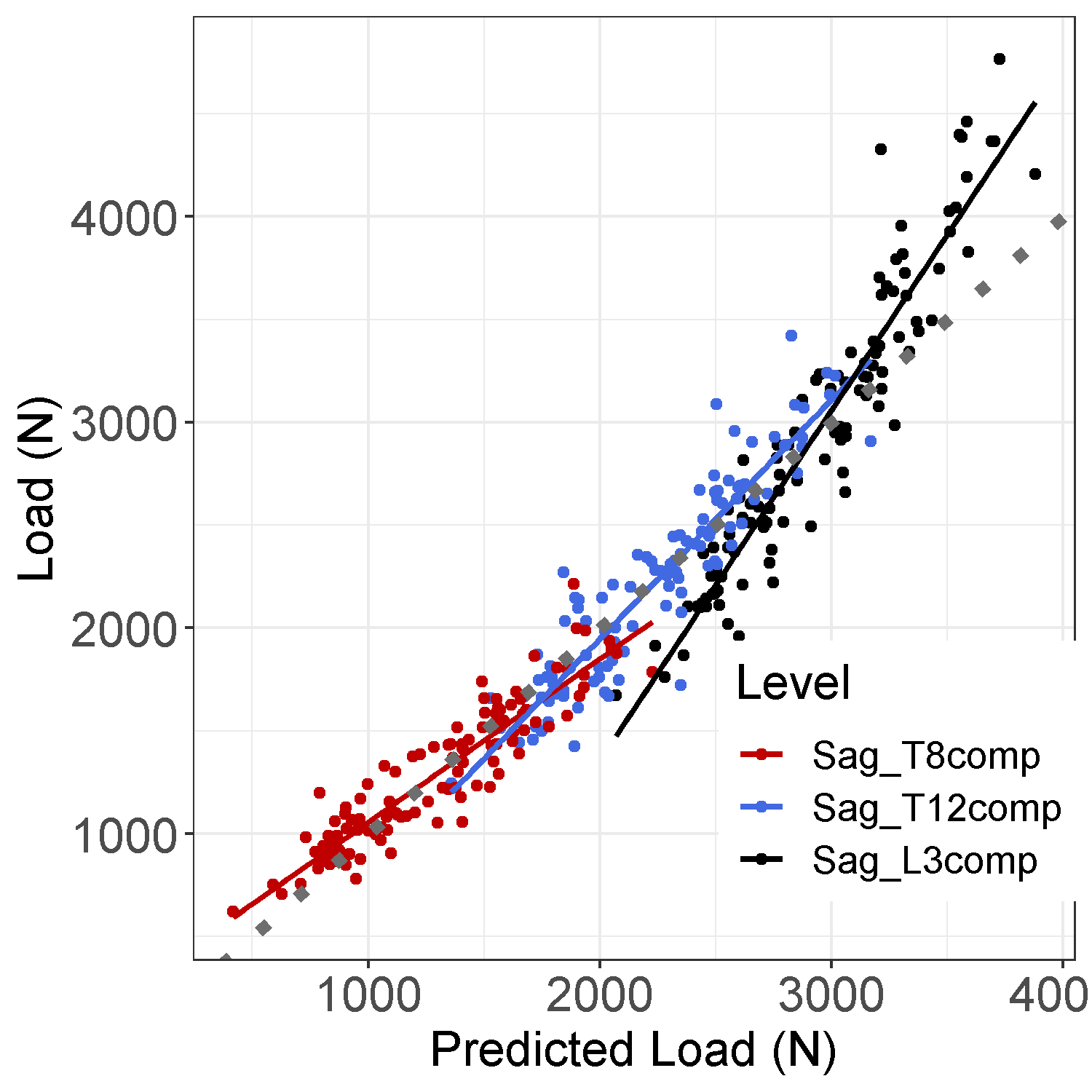

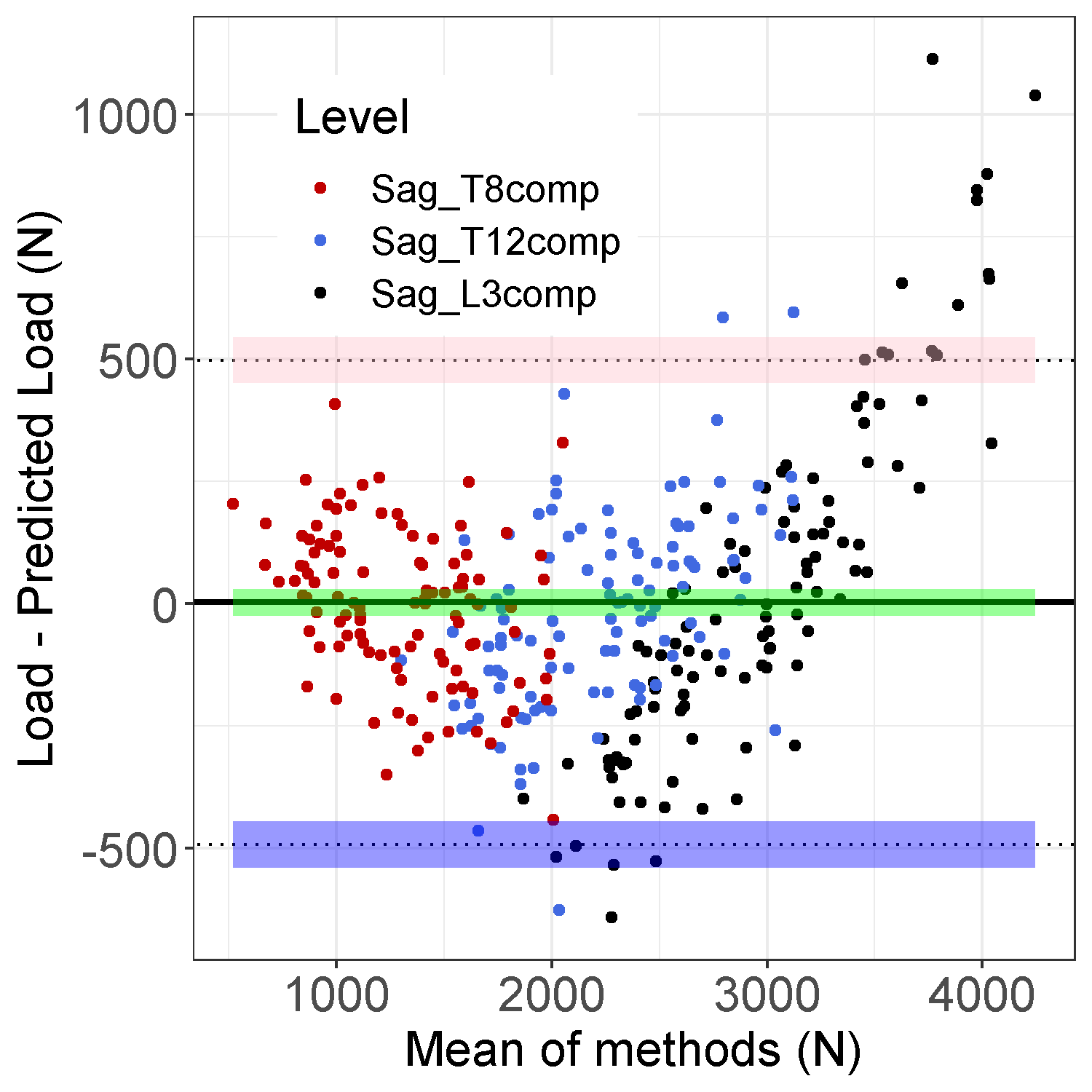


**Fig S2**: A) Predicted compressive loading vs compressive loading during flexing picking up a box with two hands, grey dot line is superimposed line of unity. B) Bland and Altman plot for prediction of compressive loading during flexing picking up a box with two hands.


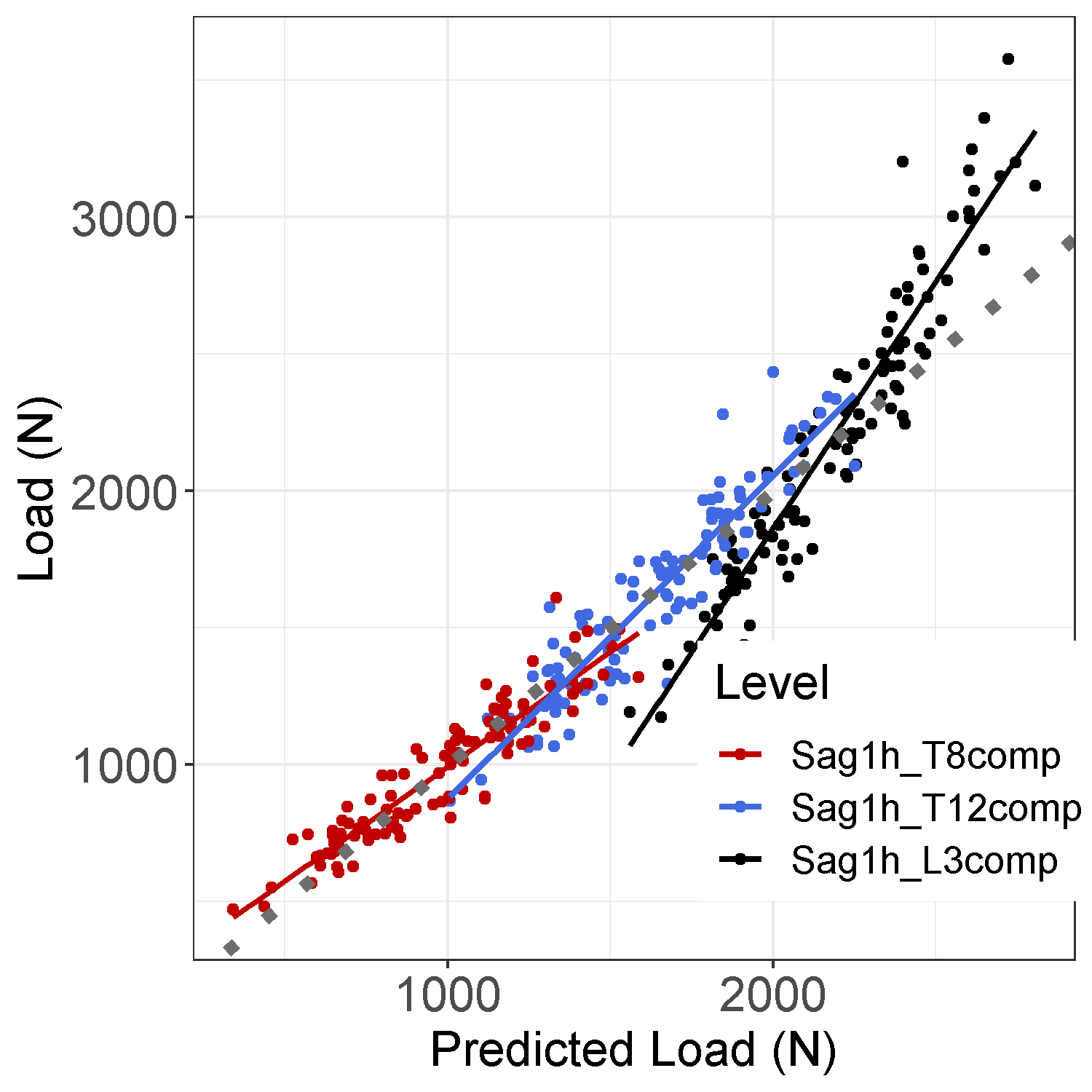

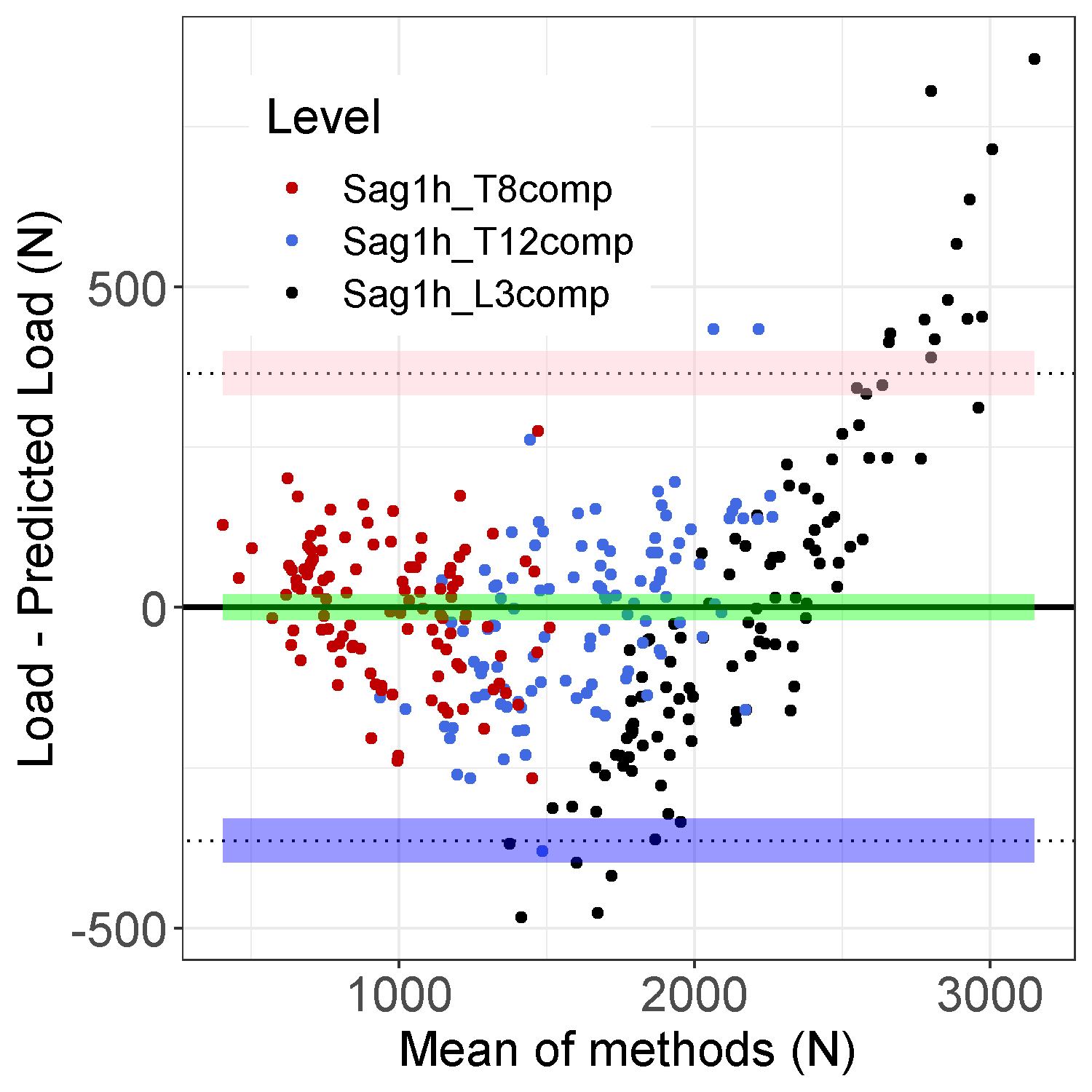


**Fig S3**: A) Predicted compressive loading vs compressive loading during flexing picking up a box with one hand, grey dot line is superimposed line of unity. B) Bland and Altman plot for prediction of compressive loading during flexing picking up a box with one hand.


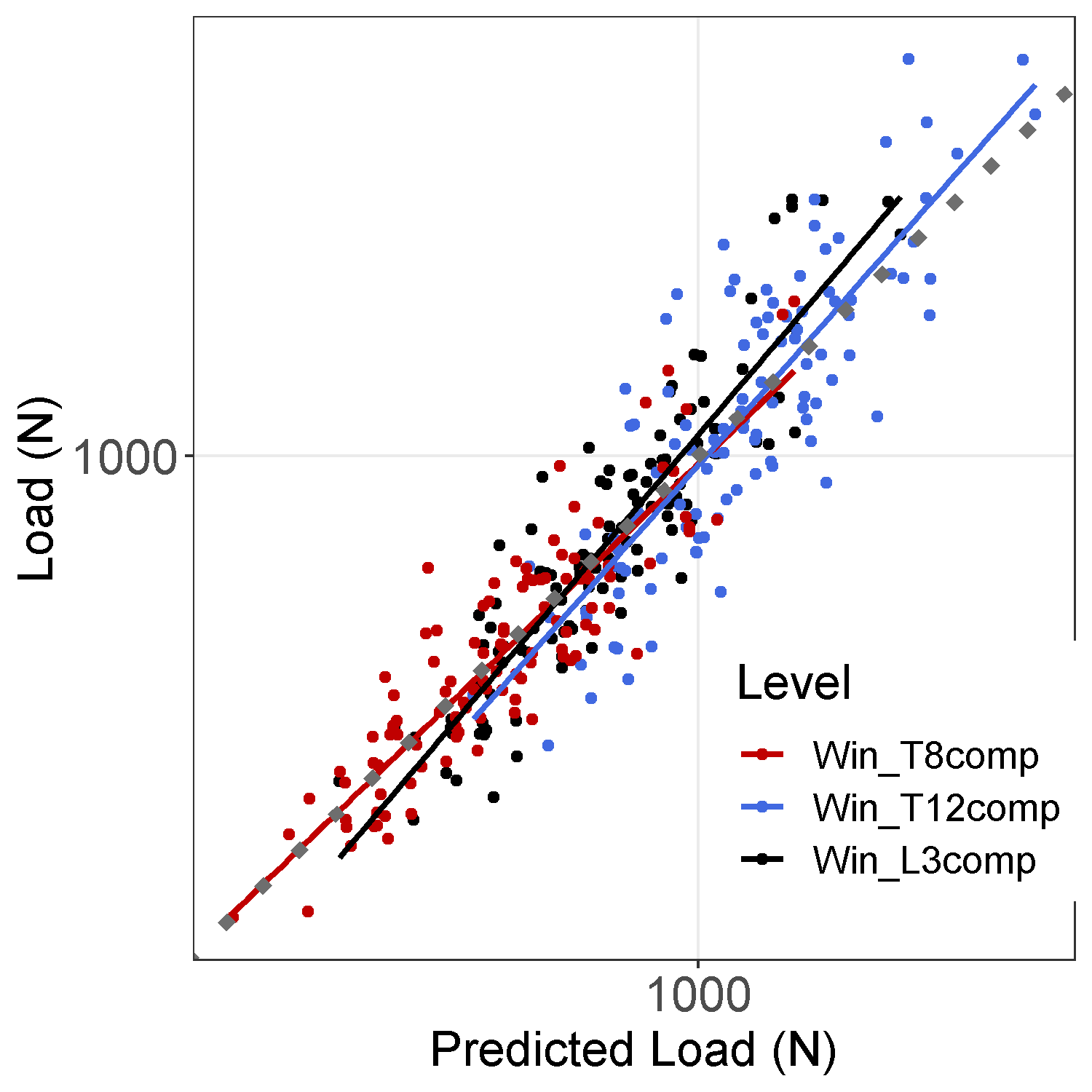

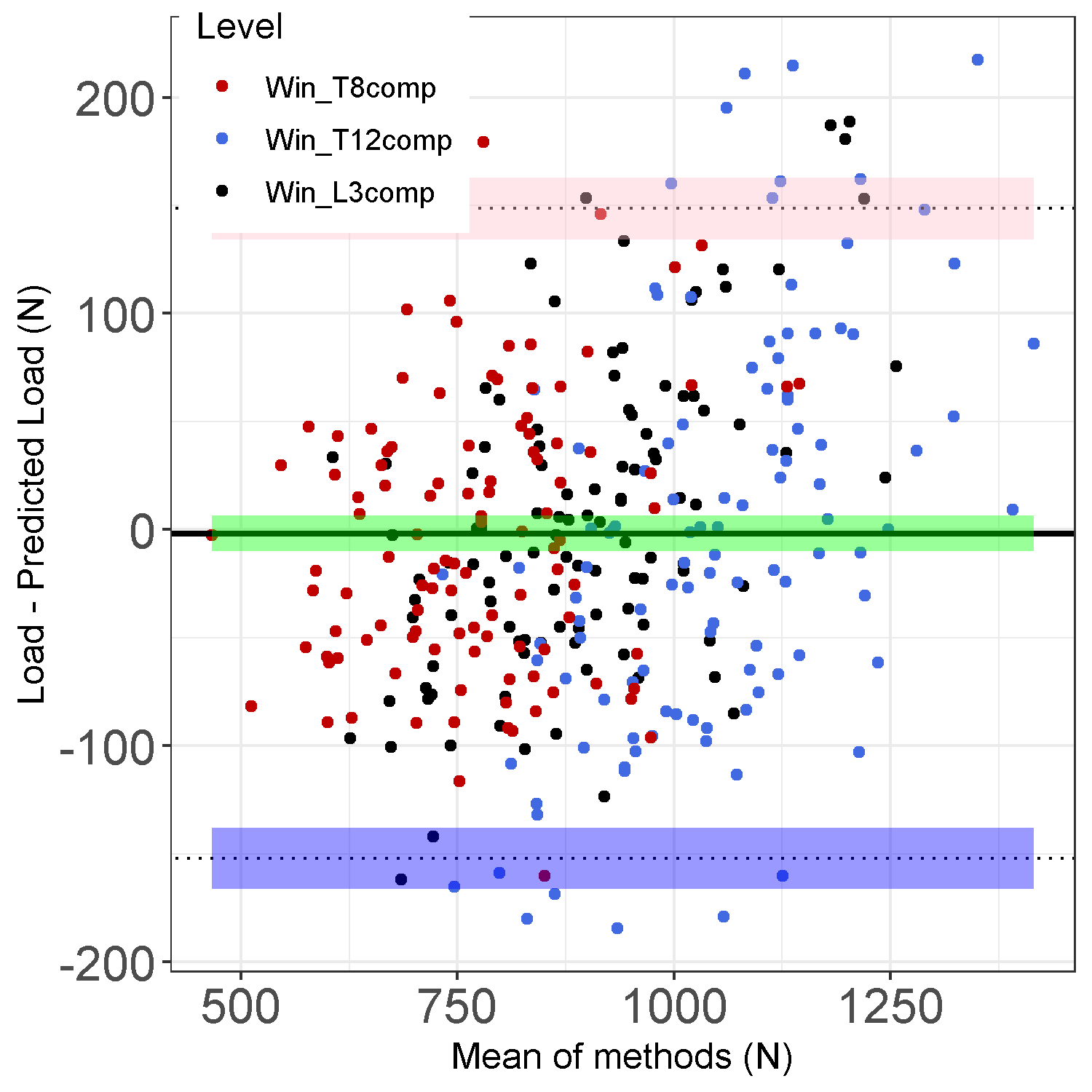


**Fig S4**: A) Predicted compressive loading vs compressive loading during opening a window, grey dot line is superimposed line of unity. B) Bland and Altman plot for prediction of compressive loading during opening a window.
